# *Leptotrichia trevisanii* Sepsis after Bone Marrow Transplantation

**DOI:** 10.3201/eid1910.121048

**Published:** 2013-10

**Authors:** Jennifer M. Schrimsher, Joseph P. McGuirk, Daniel R. Hinthorn

**Affiliations:** University of Kansas Medical Center, Kansas City, Kansas, USA (J.M. Schrimsher, J.P. McGuirk, D.R. Hinthorn);; University of Kansas Cancer Center, Kansas City, (J.P. McGuirk)

**Keywords:** Leptotrichia trevisanii, bacteria, bacteremia, sepsis, neutropenia, fever, hematopoietic stem cell transplantation, antineoplastic agents, 16S ribosomal RNA, mucositis, antibacterial drugs antibiotic prophylaxis, antimicrobial drug prophylaxis, anaerobes

**To the Editor:** *Leptotrichia* spp. have been identified as the cause of various infections. However, the most commonly reported infection is bacteremia in the setting of chemotherapy for hematologic malignancies (*1*,*2*). Only recently has *L. trevisanii* emerged as a cause of infection; case reports are rare (*3*–*5*). We recently observed 3 cases of *L. trevisanii* bacteremia in patients who had recently undergone peripheral blood stem cell transplantation (SCT.) Our goal was to identify possible causes of these infections.

The patients were 2 men and 1 woman (ages 53, 56, and 63 years, respectively) who had received myeloablative chemotherapy. The 2 men had multiple myeloma and relapsed follicular non-Hodgkin lymphoma and had neutropenic fever 5 and 4 days post-SCT, respectively. The woman had acute myelogenous leukemia that had arisen from a myelodysplastic syndrome after matched sibling donor SCT failure. She had neutropenic fever on day 13 of induction therapy.

Multiple blood cultures from >1 site (peripheral and central venous catheter [CVC] or 2 separate CVCs) obtained from each patient during the initial febrile episode grew *L. trevisanii*. For the 2 patients with positive cultures for peripheral blood and CVC sites, the peripheral culture was reported as positive before the CVC culture but not before use of the CVC. All subsequent blood cultures and catheter tip cultures from these patients had negative results for bacteria.

All organisms were cultured by using the BacT/ALERT 3D blood culture instrument (bioMeriéux, Durham, NC, USA) and standard aerobic and anaerobic media. Times to positivity were approximated (range 28–58 hours). Gram staining of isolates from culture media showed large, fusiform gram-negative rods. One isolate had gram-positive beading and was reported as gram variable. A second isolate grew anaerobically from initial subculture on 5% sheep blood agar but grew aerobically in chocolate agar in 5% CO_2_ on second subculture. A third isolate showed pinpoint growth on initial aerobic culture on sheep blood agar. No isolates were identified by using the RapID ANA II System (Remel, Lenexa, KS, USA).

One organism was identified as *Sphingomonas paucimobilis* by Vitek 2 (bioMeriéux), but this result was inconsistent with results of other biochemical tests. The 3 organisms were sent to the Mayo Medical Laboratories (Rochester, MN, USA) for anaerobic bacteria identification and speciation by 16S rRNA gene sequencing. All catheter tips were cultured by rolling a 1-inch segment of the catheter on sheep blood agar and incubating them aerobically in 5% CO_2_ for 5 days.

The reason *L. trevisanii* has only recently been identified as a cause of bacteremia in neutropenic patients is likely multifactorial. Our findings suggest routine use of 16S rRNA gene sequencing and increased numbers of bone marrow transplants as the major reasons.

*L. trevisanii* was discovered in 1999. More than a decade had passed between the availability of 16S rRNA sequencing and discovery of this bacterium. (*5*). Some authors suggested that previous lack of recognition may have been caused by fastidious growth requirements, inconsistent staining, or misidentification (*3*,*4*,*6*). No recent major changes in instrumentation, subculture algorithm, or solid media had been made before we isolated this organism, and we had not previously isolated any unidentified organisms with similar appearance and growth patterns typical of *L. trevisanii*. Unlike some species of *Leptotrichia*, *L. trevisanii* grows readily on solid media when subcultured (*3*). This finding indicates an emergent pathogen rather than a previously undiagnosed cause of bacteremia.

We have seen an increase in the number of bone marrow transplants performed, but there has been no major change in myeloablative regimens. We observed 1 case of *L. trevisanii* bacteremia in each year during 2009–2011, in which our institution performed 185, 189, and 215 transplants, respectively (overall incidence 0.5 cases/100 transplants). This finding might explain why no cases were seen previously. All 3 patients had grades 1–2 mucositis, which in the presence of neutropenia, is a known risk factor for anaerobic bacteremia in patients undergoing chemotherapy for hematologic malignancies (*3*,*7*,*8*).

Bacteremia developed in the 3 patients while they were treated with levofloxacin. The 56-year-old man responded to a cephalosporin. The 63-year-old woman did not respond to a carbapenem or vancomycin but did respond to a second carbapenem. The 53-year-old man did not respond to a cephalosporin or metronidazole but became afebrile after treatment with vancomycin. These inconsistencies did not enable us to make specific therapeutic recommendations for treatment of *L. trevisanii* infection other than to report clinical resistance to levofloxacin.

Currently recommended treatment regimens for neutropenic fever do not include treatment for anaerobic infections. Some institutions have altered treatment regimens to include antimicrobial drugs, such as meropenem, because of increases in anaerobic bacteremias (*3*,*9*). We do not believe that the number of cases of anaerobic bacteremia at our institution warrants a change in treatment policy.

On the basis of our findings, we expect an increase in the number of cases of anaerobic bacteremia after an expected increase in the number of bone marrow transplants performed. Future policies include improved treatment or prevention of mucositis, earlier detection and identification of isolates, and revision of current antimicrobial drug protocols for empiric treatment of neutropenic fever.
